# Seasonal Influenza Vaccine Uptake, Acceptance and Willingness to Vaccinate in Post-COVID-19 Vaccine Era Among Adult High-Risk Groups in Gulf Cooperation Council Countries (GCC): A Narrative Review of the Literature

**DOI:** 10.3390/vaccines14040351

**Published:** 2026-04-15

**Authors:** Moath Aljohani

**Affiliations:** Department of Family and Community Medicine, College of Medicine, Qassim University, Buraydah 52571, Saudi Arabia; m.aljohani@qu.edu.sa

**Keywords:** influenza, vaccines, epidemiology, public health, Saudi Arabia

## Abstract

Background/Objectives: Reports on seasonal influenza vaccine (SIV) coverage in Gulf Cooperation Council (GCC) countries showed lower than targeted coverage among high-risk populations both before and after the COVID-19 pandemic and subsequent COVID-19 vaccine release. This narrative review aims to synthesise SIV coverage following the introduction of COVID-19 vaccines among at-risk groups in the GCC region. Methods: Database searches included PubMed and Google Scholar for articles assessing SIV uptake, acceptance, hesitancy, and intention to vaccinate among adults in high-risk groups in GCC countries, with data collected after the introduction of COVID-19 vaccines. Results: SIV uptake ranged from 1.8% among pregnant women to 64.1% among dialysis patients in Saudi Arabia. Healthcare workers (HCWs) demonstrated the highest overall coverage, reaching 64.5% for annual uptake in Bahrain, with 79% of HCWs in Saudi Arabia intending to vaccinate. Prevalent barriers included low risk perception and consideration of influenza as a mild disease not necessitating SIV uptake, as well as vaccine effectiveness and safety concerns. Previous vaccination, physician advice, and policy or mandates for HCWs were identified as frequent facilitators of uptake. Conclusion: Suboptimal uptake was reported among most high-risk groups in GCC countries. Health Belief Model components and physician involvement appear to have a significant impact on vaccine uptake among the intended population. More emphasis should be directed toward effective risk communication and action cues methods to enhance uptake among high-risk groups. Future research is needed to cover understudied areas like the elderly aged ≥ 65 years, cancer and other high-risk groups, in addition to further studies for GCC countries other than Saudi Arabia in the post-COVID-19 vaccine period.

## 1. Introduction

Seasonal influenza is a well-known cause of respiratory viral disease and a major source of morbidity, mortality and economic loss worldwide [1]. Influenza viruses cause an acute disease within a short time after exposure, typically resulting in a self-limited disease course and recovery within a short duration [1]. However, individuals at high risk of severe disease, such as those at extremes of age (i.e., children under five years, and elderly aged ≥ 65 years), pregnant women, individuals with immunocompromised status or chronic medical conditions, are at higher risk for severe consequences like hospitalisation, Intensive care admission and the need to receive antiviral therapy [1,2].

Viral transmission occurs via droplet or touching contaminated surfaces, especially in healthcare settings, where workers may transmit the infection to vulnerable individuals [1,3]. Epidemics can occur due to seasonal influenza at any time of the year, but they peak during the winter season, while in tropical countries, they can happen throughout the year [1,4,5]. Each year, associated with a burden of 1 billion cases annually, 5 million hospitalised cases and up to 650 thousand deaths each year [1]. Moreover, influenza cases are associated with high economic and productivity costs, as they lead to increased absenteeism and greater utilisation of healthcare services. For example, the cost of attending an influenza case in the US reached 363 United States Dollars (USD), while in European countries, though lower than in the US, it constitutes a significant portion of healthcare costs. The economic burden was higher among older age groups, complicated cases and other at-risk groups [6].

Seasonal influenza in Gulf Cooperation Council (GCC) countries, including the Kingdom of Saudi Arabia, Kuwait, Bahrain, the United Arab Emirates, Qatar, and Oman, is also associated with increased burden. In Saudi Arabia, the hospitalisation and death rates were 294 and 4.7 per 100,000 population, leading to 13,982 admitted cases and more than 1500 death in the 2022–2023 season. Elevated rates noted among ≤4 years old and ≥65 years age groups [5]. The severe acute respiratory disease management cost approached 2730 million USD, with differences in cost noted for early versus delayed cases presented with complications [7]. Data from Saudi Arabia, the Emirates and other countries in the Middle East and North Africa showed that influenza vaccination is assoicated with more than two-fold lower risk of contracting influenza, combined with increased productivity days and cost savings reaching USD276.85 per individual [8].

Despite this burden, effective prevention strategies exist, with vaccination against seasonal influenza remaining the best way, as the vaccine has been shown to be safe and effective. It reduces the risk of disease development or disease severity, leading to a lower risk of hospitalisation and ICU admission [1,9]. During the 2024–2025 season, data from the US showed among adults aged ≥ 18 years, the vaccine demonstrated a protection level approaching 54% and 55% against outpatient visits and hospitalisation, respectively [9]. Additionally, some studies suggested it is associated with 34% and 45% decrease in cardiovascular adverse events and related mortality compared to non-vaccinated individuals [10,11]. Consequently, it is indicated for almost everyone aged six months and older, but with a specific focus on individuals at high risk of severe diseases and healthcare workers [12]. Vaccines are usually developed against the circulating strains each year. Most vaccines administered globally are in injectable form or inactivated or recombinant type; however, recent live attenuated vaccines that are self-administered through nasal spray have been approved in the United States (US) [13]. Other modes of prevention include strengthening hand hygiene and infection control practices [1,12].

Though the vaccine has been in existence for decades, some challenges associated with vaccine hesitancy exist. Doubts regarding the need for and effectiveness of the vaccine remained a cornerstone of influenza hesitancy in GCC countries before the COVID-19 pandemic [14]. This might also be affected by the controversy surrounding the introduction of COVID-19 vaccines worldwide. Hesitancy, defined as delay or refusal in case of vaccine availability, was considered one of the top 10 global health threats even before the pandemic, along with the threat of a global influenza pandemic [15]. Such an impact on seasonal influenza vaccine acceptance was not fully synthesised in GCC countries, which limits the capacity to apply decision-informed policy intended to increase SIV uptake in the region. GCC is a council that includes several countries, such as Saudi Arabia, Kuwait, the United Arab Emirates, Oman, Qatar and Bahrain [16]. These countries share characteristics, such as geographical proximity and language, and aim to increase coordination across sectors, including the economy, trade, education, and health affairs [16]. Because of these similarities, they are exposed to similar vaccine-related information on the internet, especially social media platforms. Such information that flooded the internet post-COVID-19 vaccine introduction may have increased hesitancy toward vaccines, even for vaccines other than COVID-19 vaccines; such misinformation was a concern even before the pandemic [17]. These challenges add to the yearly need to revaccinate with common strains of that year. This narrative review aims to synthesise the prevalence of vaccine acceptance, hesitancy, uptake, and willingness to vaccinate in GCC countries after the introduction of the COVID-19 vaccine.

## 2. Materials and Methods

**Figure 1:**
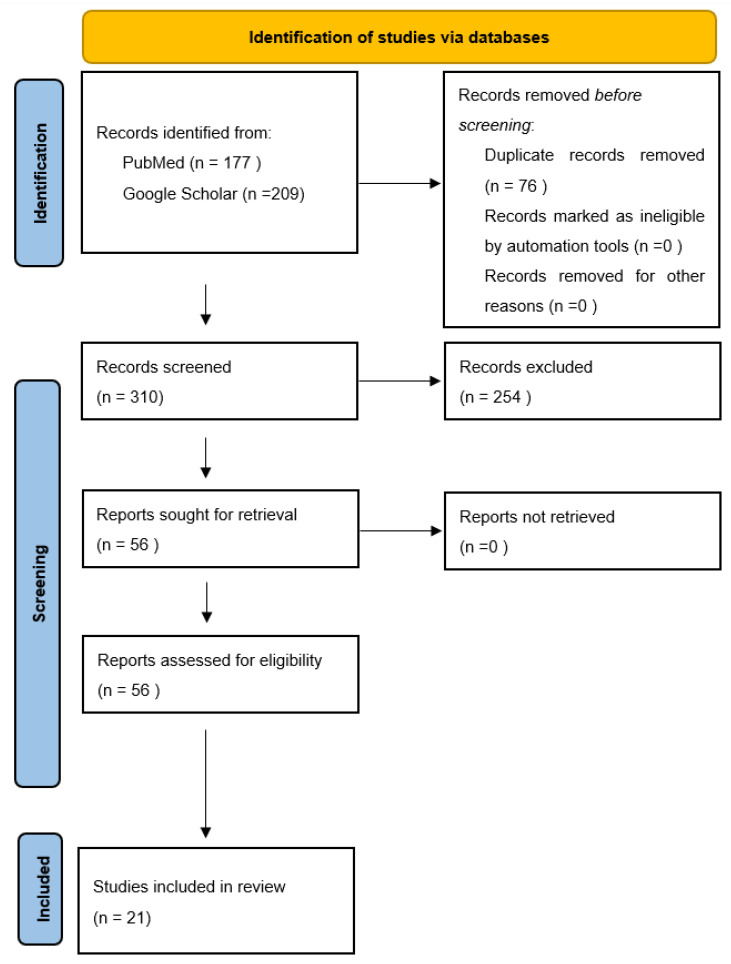
Flow diagram of article selection procedure.

This narrative review is based on studies of SIV uptake, acceptance, hesitancy and intention to SIV among adult high-risk groups investigated after the introduction of the COVID-19 vaccine in GCC countries, namely Saudi Arabia, Kuwait, Bahrain, Emirates, Qatar, and Oman. The database search included PubMed and Google Scholar. PubMed search string was as follow (“Influenza Vaccines”[Mesh] OR “influenza vaccine” OR “flu vaccine” OR “seasonal influenza vaccine”) AND (“Saudi Arabia”[Mesh] OR “United Arab Emirates”[Mesh] OR “Qatar”[Mesh] OR “Oman”[Mesh] OR “Kuwait”[Mesh] OR “Bahrain”[Mesh] OR Saudi OR “Saudi Arabia” OR Emirates OR “United Arab Emirates” OR UAE OR Qatar OR Oman OR Kuwait OR Bahrain). Results filtered to include studies published from December 2020 to December 2025. Additionally, we searched Google Scholar using similar search terms and screened the first 200 results, sorted by relevance. In order to find additional relevant articles, we added variations in common keywords related to high-risk groups such as the elderly, diabetes, cardiovascular disease, autoimmune disease, pregnancy, chronic disease and cancer, and identified an additional 9 articles. In general, the search strategy was broad to identify needed studies and was modified accordingly. If a contradiction in reporting between the text and table was identified, the tables were followed. Figure 1 shows the flow diagram representing the article extraction procedure.

Articles were included if they focused on adults considered a high-risk group for seasonal influenza and if the data collection period began in December 2020 or later, after the COVID-19 vaccine introduction. Articles were excluded if they focused on the paediatric or parent population. Studies were also excluded if they reported results from adults who were not directly part of the high-risk groups, such as healthcare college students, or from the general population that did not report SIV estimates for the elderly. For studies of the general population, estimates of SIV for the elderly were included if results on influenza vaccine status were stratified by age group (specifically, ≥50 years); otherwise, they were excluded. If the data collection timing could not be ascertained to be during the intended period for this review, they were also excluded. Literature reviews, systematic reviews, and preprints were excluded. If the questions about vaccine uptake were about the opinion of physicians regarding other high-risk groups, they were also excluded.

We encountered heterogeneity in the SIV outcome definition as some studies reported SIV uptake with a specific period of time, like “last year”, “last 12 months” or “annual adherence”, while others reported “willingness to be vaccinated during next year or flu season”, while some reported it as “ever vaccination” without specifying a year. In addition, we found no studies covering the elderly age group (≥65 years) as defined by WHO, therefore we incorporated age groups ≥ 50 years as proxy or surrogate for 65 age group when reported in general population studies and this is in line with vaccination recommendation of SIV for elderly in Saudi Arabia where the current elderly specified recommendation for SIV start at the age of 50 years [18]. Furthermore, more studies were conducted in Saudi Arabia than in other GCC countries, reflecting a larger population and a greater number of active healthcare researchers. This lack of standardised outcome combined with the paucity of studies across other GCC countries and other high-risk groups like cancer patients led to no restriction being applied on outcome definition, where all related articles of vaccination status were included in this narrative review; in line with that, these factors limited the possibility of conducting a meta-analysis or systematic review.

## 3. Results

Several studies included high-risk populations for seasonal influenza in GCC countries. The studies included (Table 1) specific populations like pregnant women, patients with kidney diseases undergoing dialysis, patients with autoimmune rheumatic diseases (AIRDs), patients with inflammatory bowel diseases, such as Crohn’s disease (CD) and ulcerative colitis (UC), patients with asthma, older adults and healthcare workers. Additional studies included samples with a collective chronic disease population. Table 2 shows the main SIV uptake predictors, barriers and facilitators for studies specifically reporting SIV acceptance, uptake or intention to vaccinate among specific high-risk groups only.

### 3.1. Pregnancy

Three studies in Saudi Arabia evaluated SIV acceptance and uptake among pregnant women in the post-COVID-19 vaccine release. Overall, these studies demonstrated marked variation and suboptimal acceptance of SIV when compared to the COVID-19 vaccine. In a large survey-based study conducted in Madinah, Saudi Arabia, including 1790 pregnant women [19]. A substantial difference in uptake between the seasonal influenza and COVID-19 vaccines was noted. Very high uptake was revealed for the COVID-19 pandemic vaccine (96.1%), while 53.7% of pregnant women reported taking a dose of SIV (*p* < 0.001) [19]. Consistent with SIV uptake, more than half of them expressed concerns about personal or family member exposure to influenza. When exploring the Health Belief Model (HBM) construct, concerns about influenza exposure and vaccine safety emerged, including the possibility that SIV may increase the likelihood of influenza susceptibility and severity, as well as persistent doubt about its effectiveness against influenza. Physicians’ advice appeared to be a strong facilitator, with the willingness to vaccinate reaching 73.2%, compared with 49.2% when family members recommended vaccination [19]. Similar findings were observed in another study, where nearly 35% of pregnant women reported previous SIV uptake, with notable barriers such as worries about injection and vaccine safety, doubt regarding vaccine necessity and negative experience with previous SIV, and only a minority (10.1%) received vaccine information from their HCWs [20].

Moreover, another study among 501 pregnant women in Saudi Arabia during 2024 reported low uptake of SIV despite the fact that more than 30% of pregnant women reported having at least one of the chronic diseases, and the majority (72%) had been pregnant before at least once [21]. Only a subset (1.8%) reported taking the influenza vaccine during the current pregnancy, in comparison to higher vaccination coverage noted with Tdap (3.2%) [21]. Similarity was observed when comparing vaccination coverage during their previous pregnancies; vaccine uptake remained low for SIV (3.7%) and Tdap (9%). As expected and consistent with the Madinah study, the strongest motivator was the recommendation of their maternity health provider for both vaccines (77.8%) or public health officials (22.2%) [21].

The top three reasons for not getting pregnancy-indicated vaccines for both influenza and Tdap were identical, such as not considering pregnant women as a high-risk group and not necessitating taking the vaccine, while others had foetal safety concerns [21]. Although the vaccines are widely available, most participants (87.4%) indicated that the public needs additional information regarding recommended vaccines during pregnancy. When testing for associated sociodemographic and obstetrical factors, younger women, healthcare workers and first-time pregnant women were associated with vaccination uptake. In contrast, obstetric-related factors such as the number of prenatal visits, the location of obstetric care, and whether the current pregnancy was planned were not associated with vaccination uptake (*p* > 0.05) [21]. Notably, among these studies, the first two studies lack clarity in the methodology section on how they asked about their uptake of SIV, whether it was in their current or previous pregnancies, or whether it was an uptake irrespective of pregnancy status [19,20]. Such details are lacking, which could explain the relatively high uptake compared to the current pregnancy uptake identified in Altheyab et al.’s study [21].

### 3.2. Kidney Diseases

Evidence from patients with chronic kidney disease undergoing dialysis showed a relatively high SIV rate despite being below the WHO target of 75% for older adults and chronic disease patients [22,40]. In a study conducted in Taif, Saudi Arabia, among 463 dialysis patients, more than 64% reported uptake of SIV during the 2022 influenza season, while fewer than half were compliant with annual vaccination. A considerable percentage were non-compliant with annual vaccination or never had an SIV, indicating vaccine hesitancy exists even in well-established high-risk groups who have frequent encounters with the healthcare system [22]. Among the reasons for non-vaccination during the study year were concerns related to vaccine safety, doubts regarding vaccine effectiveness, and worries that the vaccine may worsen their kidney disease [22]. The most reported source of SIV information was conventional methods, such as TV and radio, by three-quarters of dialysis patients, followed by social media channels. Involvement of HCWs in SIV recommendations was frequent; a substantial portion (82.3%) received SIV recommendations from the treating nephrologist, and a smaller number from general physicians. Interestingly, some patients reported a proactive behaviour by asking their HCWs about SIV [22].

Across analyses, factors related to influenza knowledge, perceived severity and health-seeking behaviours appeared to be significantly associated with improved SIV compliance. Good level of influenza-related knowledge (OR = 2.1, 95% CI 1.4–3.2), higher perceived risk of death (OR = 2.6, 95% CI 1.6–4.3), proactive behaviours by asking healthcare workers, including general physicians (OR = 2.3, 95% CI 1.2–4.3) and nephrologists (OR = 2.0, 95% CI 1.2–3.4). Relying on traditional media as a source for vaccine information has been shown to be a significant predictor of SIV among dialysis patients [22]. Overall, these data imply that even with the high coverage of dialysis patients compared to other chronic disease patients, gaps in disease severity perception and HCW involvement still exist, necessitating targeted programmes focused on patient-sustained education and proactive engagement and promotion of vaccines by HCWs during patient encounters.

### 3.3. Autoimmune Diseases

Studies on SIV uptake among the Saudi population with autoimmune diseases suggest a moderate level of acceptance in addition to persistent knowledge and behavioural barriers post-COVID-19 vaccine introduction. Among 219 individuals aged ≥ 14 years attending autoimmune rheumatic diseases (AIRDs) clinics in 2024, the majority (53.4%) received the influenza vaccine, with higher uptake among males; while for pneumococcal and herpes vaccines, the figures were lower, at 42.9% and 24.2%, respectively. The sample included patients diagnosed with different autoimmune diseases, including rheumatoid arthritis (RA), and systemic lupus erythematosus (SLE) in addition to patients with Sjogren’s syndrome, ankylosing spondylitis, and other diseases characterised by dysfunctional immune reactions [23]. Nevertheless, the study methodology did not report whether that uptake was during the most recent influenza season or other seasons, though the authors cross-checked the vaccination data with the participants’ health records [23]. Among the 91 (41.6%) who did not receive any vaccine, the most reported challenges were time limitations and doubts about vaccine safety. An additional subset of patients lacked a physician recommendation or perceived SIV as a contraindicated medication [23]. When studying vaccination predictors among AIRDs patients, the current use of biological Disease Modifying Antirheumatic Drugs (DMARDs) appeared to be strongly significant compared to non-users. Among corticosteroid users, recent use (1 month or less) was shown to increase the probability of vaccination compared to longer use (>1 year) [23]. It is worth noting that these barriers and associations were not specifically measured for the influenza vaccine. Therefore, the true association might have been masked or overestimated for such variables and their association with influenza vaccine uptake.

Additional data were reported among 261 patients following up at rheumatology and SLE clinics in Saudi Arabia between November 2024 and February 2025 [24]. Out of the total, 50.2% reported previous SIV vaccination, while only 27.6% reported SIV in the last two years [24]. More than half (57.1%) of respondents cited being informed by their physicians to vaccinate. A similar proportion believed that SIV was effective and safe, as well as the superiority of SIV in preventing influenza-related complications. Although most patients were knowledgeable about the facts that SIV is recommended for individuals with chronic disease, nearly one-quarter knew that pregnancy is not a contraindication for SIV. Additional knowledge gaps and misconceptions were exhibited, such as the 25% believed that SIV can hinder the immune system, and 45.6% believed that traditional therapies and herbs are better to be used than SIV. Almost all respondents have knowledge of SIV being freely provided in Saudi Arabia [24]. Vaccinated patients were older (*p* < 0.046), more likely to affirm the safety and effectiveness of the SIV (*p* < 0.001), and confirm that vaccination is the best way to avoid influenza-associated complications (*p* < 0.001). Those who considered SIV as recommended for chronic disease patients (*p* < 0.001) and informed by a doctor (*p* < 0.015) also showed higher SIV uptake rates.

Regarding other GCC countries, the finding among 394 Inflammatory bowel disease patients in Kuwait, including both ulcerative colitis and Crohn’s disease patients, adhering to their prescribed biologic medication, where data collected during 2022 and 2023 showed suboptimal uptake, as only 20% received the SIV [25]. In relation to adhering to a list of recommended vaccines, including Measles, Mumps and Rubella, Herpes Zoster, Hepatitis A, Hepatitis B, COVID-19, Diphtheria, Tetanus and Pertussis containing vaccines, pneumococcal, meningococcal, Hemophilus influenzae type B, seasonal influenza and other vaccines. There was no significant association across age groups, whether having a diagnosis of Crohn’s disease or ulcerative colitis, or nationality; *p* ≥ 0.05. Only sex emerged as a significant predictor were females showed higher adherence rates (79.9%) to all recommended vaccines compared to males (46.4%), *p* = 0.001 [25].

### 3.4. Asthma

Only one study assessing the SIV acceptance among asthmatic patients in Saudi Arabia during the post-COVID-19 vaccine rollout was extracted. The study conducted among adult asthmatic patients attending primary care centres in Riyadh extracted a total of 2689 electronic health records from Raqeem, a nation-level electronic health records system that covers primary healthcare services in Saudi Arabia. Vaccination data were acquired from the Sehhaty app [26]. Of the screened records, only 18.67% had the SIV during the 2023–2024 season compared to 0.26% and 93.1% for pneumococcal and COVID-19 vaccines, respectively. Asthmatic patients aged 50–64 had higher (24%) vaccination rates compared to younger or older age groups (17% and 19%, respectively) (*p* = 0.002). Other vaccine uptake, such as the pneumococcal vaccine (*p* = 0.026) and the COVID-19 vaccine (*p* = 0.001), demonstrated a significant association with SIV uptake. Having prescribed a high dose of corticosteroid showed a significant relationship with SIV uptake as well (*p* = 0.001) [26].

### 3.5. Diabetes Mellitus

Data from a cross-sectional study conducted in Bahrain among 393 diabetic patients who attended primary healthcare centres in February 2022 showed that nearly half (50.6%) have had the SIV [27]. The primary factor influencing positive vaccination practices was physician advice (72.9% of vaccinators), while the most reported barrier among non-vaccinators was the perception of influenza as a mild disease (40.2%). More than half (58.3%) of diabetics considered influenza symptoms tend to be worse with diabetes, and 44.8% had the conviction that influenza increases the risk of severe consequences, such as poor glycaemic control, pneumonia and hospital admissions. More than 60% believed it protects against serious influenza complications and acknowledged the importance of annual vaccine uptake [27]. In general, 31% of respondents displayed a good level of knowledge regarding influenza and SIV, and 65.1% demonstrated an encouraging attitude toward flu vaccination. Higher SIV uptake was noted among females, attainers of an intermediate level of education, and who lived longer with diabetes (OR = 2; *p* = 0.018). Having a good level of knowledge regarding influenza and SIV was also associated with increased odds of uptake by 2.9, (*p* < 0.001). A limitation of this study is that the author did not specify which year the patients received their influenza vaccine; it is mentioned as “previously,” without specifying a year or season, limiting the identification of the timing of uptake as post-COVID-19 vaccine [27].

Additional findings from a multicenter EHR review of diabetic patients attending care-specific centres on their adherence to Advisory Committee on Immunization Practices show that 34.83% out of 709 have been vaccinated with the annual influenza vaccine, with lack of information on vaccines’ advantages and side effects being frequently cited barriers [28]. Moreover, when diabetics were inquired about vaccination history of seasonal influenza, COVID-19, and pneumococcal vaccines, the highest vaccination rates were reported for COVID-19 (88.7%), and influenza 60.8%, compared to 27.1% for pneumococcal vaccines [29]. The majority of diabetics believed that they should receive SIV. This agreement level was higher than that of COVID-19 and pneumococcal vaccines. Out of them, 72% reported knowledge of the need to take the vaccine annually, and 45.6% stated a healthcare professional as the main source of flu-related information, while 51.8% considered the SIV effective in some way, and the rest were not knowledgeable or sceptical toward vaccine efficacy. The most frequently reported barrier to respiratory disease vaccination was the conviction that vaccines are harmful or ineffective. Others included concerns related to vaccine ingredients or their necessity [29].

### 3.6. Grouped Chronic Diseases

Evidence from various GCC countries showed inconsistent SIV uptake among chronic disease patients post COVID-19 vaccine introduction. Differences were obvious in annual and lifetime uptakes. In a study conducted in 2022 in the form of an online survey [30]. Out of the total 825 participants from Jazan, Saudi Arabia. About 249 (30%) of the participants had a diagnosis of at least one chronic condition, with hypertension, diabetes, asthma and sickle cell disease being the most reported. Among chronic disease patients in the region, 58.6% reported a history of SIV, and only 17.3% of chronic disease patients received the vaccine on an annual basis [30].

Further data from an online survey conducted in the last quarter of 2023, involving 241 participants with at least one chronic disease [31]. During the study influenza season, 43.2% reported either taking SIV or had the intention to vaccinate, while 10.8% reported at least one episode of SIV uptake during the previous COVID-19 pandemic flu seasons (2020–2022). The most reported diseases were 29% DM, 28.2% asthma, 23.2% hypertension and 18.3% for obesity. When examining factors associated with SIV uptake, no significant relationships were noted among age, gender, marital status, educational attainment, occupational status or the number of chronic diseases (*p* > 0.05). However, receiving information from untrusted sources had strong negative impact of SIV coverage, as they were more likely to delay or refuse the SIV (*p* < 0.001) while when evaluating the association of clinical history and previous SIV during the pandemic years with SIV during the current season of the study, immunodeficiency and DM patients showed opposite responses were immunocompromised individuals were three times more SIV hesitant than immunocompetent individuals (*p* = 0.03) while DM patients were less likely to be vaccine hesitant (*p* = 0.03). SIV history during the pandemic was linked to vaccine acceptance (OR = 0.23, 95% CI 0.095–0.55) [31]. Among vaccine acceptors (n = 104), healthcare professional advice was the most frequent motivator (29.8%), followed by concern about personal health situation (20.2%), being prone to recurrent respiratory infections or colds (15.4%), and easy accessibility to vaccination sites (10.6%). In contrast, non-vaccinated participants (n = 137), the most commonly cited reasons for refusal were being uncomfortable with SIV for no particular reason, doubts about vaccine effectiveness, inadequacy of vaccine information, a lack of a sense of vaccine necessity, and fear of vaccine side effects. Vaccine accessibility was not an issue, as only 2.2% reported that vaccination centres were not available nearby [31].

Additional findings form hospital based study in Oman, where data were collected during 2023 and 2024 from 158 admitted patients at a tertiary care hospital, including elderly (≥65 years old) and adults indicated for influenza vaccination, as they have high-risk chronic medical conditions, in order to assess policy adherence of vaccinating admitted individuals upon discharge [32]. The data showed very low uptake, with only 4.4% receiving the seasonal influenza vaccine. The methodology did not specify the period between their recent admission and their uptake assessment.

### 3.7. Older Adults

Few studies in the region evaluated vaccine uptake specifically among the elderly in the region. Therefore, in this review, we extracted studies assessing SIV uptake among the elderly aged ≥ 50 years, as a proxy for the WHO’s commonly cited high-risk group (≥65 years). In addition to that, in some GCC countries, the indication for the elderly is starting from the age of 50, like in Saudi Arabia [1,18]. Studies aimed to assess seasonal influenza and vaccine awareness in Saudi Arabia revealed suboptimal uptake among older adults. A survey-based study conducted in the Asser region between December 2020 and January 2021 among 386 adults aged ≥ 60 years, with a mean age of 72.3 [33]. A significant portion of the study population reported a high prevalence of chronic diseases, including DM (78%), hypertension (71%), heart disease, and asthma or Chronic Obstructive Pulmonary Disease COPD. Vaccine awareness was very high: 91% knew SIV existed, the majority believed it was safe, and recognised its role in protecting against influenza infection and related complications. Overall, 43.3% had good awareness of the SIV, while 57.3% of them demostrated good awareness of both seasonal influenza and its vaccine [33].

Regarding vaccine uptake, 66.6% reported receiving the vaccine during the study year, and more than 35% were regular recipients of SIV over the last five years. Among vaccinated elderly, doctors were the most frequent source of SIV advice (59.1%), followed by advice encountered through social media (14.4%) and from friends and family (14%). Interestingly, 12.5% reported it was their own deliberate decision. For non-vaccinated elderly, the conviction that flu is a mild disease, thereby not necessitating vaccination, was the most stated obstacle for vaccination (34.1%), followed by questioning vaccine effectiveness in infection prevention (28.7%), 15.5% stated having an alternative form of prevention, and only 8.5% feared a serious side effect from SIV [33].

Regarding the factors associated with regular uptake over the last five years, these include older married males and residing in rural areas, compared with females; divorced/widowed living in urban areas; and attainers of secondary school, compared with lower levels of education. Living with a partner and higher socioeconomic status were associated with better vaccine coverage (*p* = 0.016 and *p* = 0.006, respectively). Higher uptake, though not significant, was also noted among older adults with kidney diseases, hypertension and diabetes. Additionally, among the 19% who had a history of hospital admission because of influenza, they reported regular uptake compared to those without a history of admission; *p* = 0.049 [33].

Another study from Saudi Arabia included 148 adults aged 51 years, who revealed that nearly 42% had been vaccinated or intended to vaccinate during the 2023 season [34]. An additional study assessed the predictors and prevalence of SIV post-COVID-19 [35]. The study sample included 33 individuals aged ≥ 55 years, representing less than 5% of the sample. Only 18.2% reported SIV uptake during the preceding 12 months, ranking second lowest after the youngest age group, 18–24 (18%). Overall, the total sample exhibited higher uptake among males, aged 25–54 years, compared to those aged ≥ 55 years. Healthcare practitioners and chronic disease patients also had higher odds of SIV uptake [35].

### 3.8. Healthcare Workers

Across GCC countries, HCWs had higher SIV acceptance compared to most other high-risk individuals, although the level of acceptance varied from one study to another according to country, work type and the timing of assessment. Data collected through online platforms from 190 family physicians in Qatar in October 2021 revealed a high lifetime 96.3% uptake [36]. Along with 73.3% being vaccinated during the last season prior to the study data collection. The vaccine uptake was noted to be higher among males (*p* = 0.002) and perfect uptake among those aged 65 years and more (100%), contrasting (45.5%) among the youngest included age group (22–43 years), *p* = 0.004. The trend was almost incremental, with uptake increasing across older age groups and work experience (*p* = 0.005) in primary care centres. Living with high-risk groups was not associated with a significant increase in vaccination rates (*p* > 0.05) [36]. Almost universal knowledge of SIV-free provision by the state’s MoH and the vaccine recommendation, including the specified high-risk individuals. A significant number of HCWs had negative experiences with the vaccine as a result of vaccine-associated side effects, and others find it difficult to take the vaccine every year. The striking majority of physicians had a strong belief that they can get influenza even if they are up-to-date with flu vaccination, though they acknowledge its role in protecting close contacts, society and the healthcare system [36]. Of note, male physicians with chronic diseases who received the vaccine during the last or any influenza season and those who had received more previous vaccines reported fewer barriers against vaccination. Borderline significance noted among doctors who received five or more SIV, as they had a better perception of vaccine benefits compared to those with a lower number of doses [36].

HCWs in Bahrain were surveyed online and in person during July 2024. Out of 552 participants, nursing staff constituted 45.7%, together with physicians (36.2%), forming the largest proportion of professions among the study sample [37]. In terms of practice, nearly 65% reported adherence to yearly SIV, while 16.8% never received the SIV. Almost three-quarters of HCWs were labelled as low-non-hesitant, compared with the rest, who were judged to have a moderate to high level of hesitancy toward vaccination. The majority supported vaccination mandates and follow-up on occupational vaccination status in general. Across a list of recommended vaccines for HCWS, SIV scored the highest approval, with 82.9% supporting its receipt compared with other recommended vaccines. When various sociodemographic and occupational factors were examined for their association with general vaccine hesitancy, elevated hesitancy was observed among workers in governmental hospitals compared to PHCs, and non-convinced HCWs of vaccine importance. While being local Bahraini workers and holding a positive attitude toward vaccination with regard to effectiveness, prevention of infection-associated complications and epidemic, as well as aiding in community health promotion, was associated with lower hesitancy scores [37].

Findings from Saudi Arabia in 2021, where participants were asked about their previous vaccination and their intention to vaccinate in the 2021 season, displayed an increased willingness to vaccinate compared with vaccination uptake in previous and pre-pandemic seasons. Out of 424 nurses and doctors, a higher percentage (79%) expressed willingness to vaccinate during the 2021 season, compared with 59% in 2020 and 62% in 2019 [38]. Examining the predictors of vaccination intent through adjusted analysis, older age (>40 years), females, and non-Saudis displayed a significant increase in vaccination intent. Regarding profession type, nurses exhibited three times greater intent than doctors (AOR:3.12, 95% CI 1.69–5.56; *p* < 0.001) [38]. Education, presence of comorbidities, confidence in MOH, knowledge of vaccine efficacy duration, and history of COVID-19 infection were not significant predictors of vaccination intent (*p* > 0.05).

Another Saudi study during the last quarter of 2021, after most of the population in the kingdom received one dose of the COVID-19 vaccine, reported that half (50.8%) of the 1273 participants had received the vaccine during the past 12 months, and more than 85% reported a positive vaccination history over the last five years [39,41]. When analysing the highest uptake over the past 12 months in contrast to the past five years, healthcare practitioners aged 60 years and older had 85.7% vaccination coverage, reaching 100% in the previous five years. Across various occupations, higher uptake was observed among doctors and nurses, as 55% reported vaccination during the past year, which was higher than other workers, while laboratory workers showed rates as low as 40.0%. Similarities were observed regarding vaccination status over the preceding five years. Previous vaccination over the last five years increases the odds of getting the vaccine in the previous 12 months by 2.19 (95% CI, 1.49–3.22; *p* < 0.001), and demonstrating positive attitudes in respect to SIV was 1.51 (95% CI, 1.09–2.09; *p* = 0.014) more likely to have the vaccine compared to HCWs with a negative attitude. Additional associations with improved vaccination over the past 12 months were also observed, including improved accessibility to the vaccine in the workplace, knowledge of influenza vaccine recommendations for HCWs, involvement in vaccine-related educational activities over the previous year, and working at the MOH head office compared to working in the health directorate. In contrast, having a lower perception of influenza risk that does not necessitate vaccination was revealed to be aligned with lower vaccination coverage with OR= 0.53 (95% CI, 0.32–0.86; *p* = 0.011) [39].

## 4. Discussion

This review highlights that, though wide and free provision of SIV in GCC countries, vaccine acceptance and uptake remain suboptimal across most high-risk groups post-COVID-19 vaccine introduction. This observation was consistent across included studies of at risk-groups of influenza with varying degrees of uptake and acceptance, where the highest uptake was noted among HCWs and patients undergoing dialysis [22,36,38,39]. Despite these observed levels, most high-risk groups were further away from the WHO target of 75% and as low as 1.8% among pregnant women in Saudi Arabia [40].

The HBM components emerged as consistent determinants among susceptible populations, such as low-perceived infection risk, disease severity, and lack of confidence in vaccine effectiveness, safety, or fear of injections, which were common barriers against vaccination [19,20,22,31]. In contrast, high perceived disease susceptibility, and the aim to protect self and others, along with improved accessibility associated with higher vaccination rates, confirm the effect of HBM components on vaccination tendency [19,34,36]. Among different high-risk categories, such as pregnant women and chronic disease populations, trusted sources of influenza vaccine information, such as those received from healthcare professionals, appeared to be consistent prompts for vaccination among non-HCWs in line with other influenza vaccine coverage reports [24,34,42,43,44,45,46]. These findings highlight the need to address vaccine literacy, improve accessibility and emphasise physician risk communication for target groups during encounters with the healthcare system [45]. Additional methods were suggested to improve uptake and physician engagement with at-risk groups, including reminders of vaccination and training sessions for physicians on the diseases intended for vaccination [47]. The role of the HBM construct was noticeable when comparing COVID-19 vaccine uptake to SIV, where COVID-19 vaccinated individuals tend to perceive seasonal influenza as a mild disease in contrast to COVID-19 [48].

Additional investigation of HBM’s role showed that a better understanding of influenza, its vaccine, and recommendations, combined with previous vaccination against SIV or other indicated vaccines, such as COVID-19 and pneumococcal, has been associated with increased coverage. This could be a result of enhanced self-efficacy, as prior positive experiences increase the probability of future health-seeking behaviours, as observed among the Chinese elderly [22,24,34,36,39,49]. Consistent with studies among GGC countries, the association of HBM constructs as a whole or part was exhibited across various high-risk groups, such as HCWs [50,51,52], older adults [53], pregnant women [54], and kidney recipient patients [55]. A better understanding of HBM can aid physicians in identifying the psychological determinants contributing to hesitancy to improve vaccine coverage [56].

Sociodemographic predictors include age, sex, educational attainment and economic status, which demonstrated inconsistent association with SIV acceptance and uptake. In some cases, different age groups [21,36,38], gender [36,38], with the presence of comorbidities [30,34] and working in the health sector [21] showed a higher tendency for SIV acceptance post-COVID-19 vaccine introduction; this tendency was not constant across studies in this review. Clinical factors exhibited an impact on vaccination status, among autoimmune disease patients; biologic DMARDs use among AIRDs patients was accompanied by increased uptake of several indicated vaccines in line with the pattern noted among corticosteroid asthmatic users [23,26]. Additionally, a longer duration of living with the diseases has been shown to be associated with higher vaccination rates among diabetics [57]. Data from the Italian cohort revealed elevated SIV uptake among older females with a higher number of comorbid conditions, such as cardiovascular and renal diseases [58]. The association between older age and higher prevalence of chronic diseases could be explained by increased exposure to influenza-related information and recent exposure to the healthcare system, increasing the odds of vaccination [59]. While the impact of gender on vaccination remains variable among studies, the role of social support is more consistent, as vaccination rates are noted to be higher among married individuals in the Italian cohort and the Saudi elderly, in addition to being motivated by family advice or protection among HCWs [19,58,60].

Recent data from international coverage reports of the elderly highlight higher coverage rates than those in available GCC data, although only Denmark from the European region exceeded the WHO coverage target for older adults, while Ireland and Portugal, in addition to Canada, approached the optimum level [53,61]. This level of uptake was not consistent across other countries. As of the 2024–2025 influenza season, median coverage for European countries was 47%, which reflects suboptimal coverage across most countries in the European region [61].

Vaccination rates for chronic disease also remain at a suboptimal level. In comparison, Canada, has a near-target coverage rate for seniors, while the coverage for Individuals below 65 years with chronic diseases remains at 44% during the 2023–2024 season [53]. While in Europe, it ranged from 25% to 30% [61]. It is worth noting that our review included studies with different groupings, as chronic disease studies were not specified for those aged 65 or younger.

For HCWs, the GCC rates exceeded 50% with varied coverage depending on the country and setting, in comparison to findings from meta-analysis incuded studies across several countires and periods, reported vaccination rates of 41.7% and 42.5%, overall and in Europe; in respective order, while Middle Eastern studies reported 51.3% coverage rate. In subgroup analysis, the overall coverage in 2020 onwards showed the highest coverage 52.8% compared with previous periods, notable differences exsisted in vaccination coverage according to the economic status of the included countries [50]. As observed in the current review, work-related factors, such as the type of profession, higher numbers of years spent working, and workplace free provision of SIV, were associated with more coverage [36,38,39,50]. Work policies and mandates play an important role and may serve as cues to vaccination. In Australia, vaccine coverage increased after mandates were implemented to reach 93.6%, in addition to reports from Emirates and Bahrain, where participants affirmed the role of policy requirement and administrative follow-up on vaccination status as strong facilitators for vaccination [37,60,62]. These data highlight the need to implement or reinforce mandates and policies to enhance SIV coverage in healthcare settings across Gulf countries.

Vaccination coverage showed notable variability before and after the introduction of COVID-19 vaccines, with inconsistent changes. For example, Canada witnessed a transient decline in the 2021–2022 season, then vaccination rates defaulted to pre-pandemic rates [63], while in Saudi Arabia, a meta-analysis showed coverage rates varied by population, where the general population showed drops in rates following the pandemic, contradicting the observed trends of HCWs [64]. In the US, HCWs remained at a similar level to the peak observed in the 2018–2019 season, with notable declines in the 2019 and 2020 seasons; these rates remained above the target coverage [65]. While in Italy, rates heightened following the COVID-19 pandemic, but then defaulted to pre-pandemic levels [66]. These increases were in part due to increased perceptions of respiratory disease severity and vaccination accessibility, while the later decline is proposed to result from vaccine fatigue and declining risk perception [66].

Co-administration of the indicated vaccine has proven to be an excellent facilitator for vaccination and is being adopted by more countries. Combined administration of SIV with the COVID-19 vaccine was noted among 71% of flu vaccinators in Canada [53,66]. Several reports emphasised the safety and beneficial impact of combined administration of flu vaccines with COVID-19, Respiratory Syncytial Virus, and pneumococcal vaccines, with no serious safety events or significant impact on immunogenicity reported. These characteristics were combined with increased vaccination coverage and fewer vaccine-related consultations [67,68]. Improving convenience and accessibility by providing vaccination sites other than hospitals and in the form of open day events at the workplace environment was also noted to boost vaccination among HCWs and other risk groups [31,39,63,66].

Commonly administered SIV methods include injectable or nasal spray formulations matched to at least three circulating strains during each season. However, the current methods face challenges related to SIV efficacy, matching with circulating strains, and the need for seasonal revaccination [69]. Emerging technologies have the potential to show progress, like nanotechnology applications in vaccine development, which showed success during COVID-19 vaccine development [69,70]. Additional ongoing efforts to develop a universal influenza vaccine that is effective against all flu strains, able to overcome antigenic shift and drift, and offers long-lasting protection. These efforts are showing some progress with the use of injectable antivirals like zanamivir as prophylaxis [71].

All GCC countries provide influenza vaccination free of charge for all high-risk groups [72]. For example, in Saudi Arabia, SIV is available in all MOH and other governmental primary healthcare centres around the kingdom, facilitating the vaccine accessibility where SIV appointment booking can be made through digital channels like the Sehhaty app [18]. This was reflected by studies from the region [31,34,36] citing accessibility as a motivator rather than a vaccination barrier. Unfortunately, these efforts met with suboptimal coverage. Public health authorities in GCC need to address specific determinants of SIV hesitancy, including vaccine safety, efficacy, and low perception of severity [14], and targeted recommendations for high-risk groups through different channels or during their healthcare system encounters. Misleading information regarding the vaccination safety has gained a boost after the COVID-19 vaccine release [73,74]. These efforts may oppose the vaccination attempts for infections other than COVID-19, as vaccine conspiracy observed to be associated with negative attitudes toward SIV and COVID-19, resulting in heightened vaccine safety concerns after the experiences with the COVID-19 vaccine [75]. Dynamic observation of trending safety concerns needs to be implemented by health authorities to overcome these challenges in a timely manner.

### 4.1. Limitations

This review faced several limitations, some of which are related to methodological challenges noted in GCC studies; one is the heterogeneity of study methodologies regarding sampling and surveying participants on SIV acceptance or uptake. Many studies did not measure vaccine uptake during a specific period, like during last season or past 12 months, in contrast some had extended or not specified period for uptake evaluation like “did you receive seasonal flu vaccine”, “ever received flu vaccine” or “last 2 season uptake” which may overestimated the actual vaccine acceptance and more prone to recall issues. This heterogeneity affects the comparability of different high-risk populations and countries. Additionally, few studies matched participant uptake with EHR data, which further hinders the accuracy of vaccine timing or actual uptake. In line with recall issue encountered from individual studies, several studies were based non-probability sampling on an online route for data collection, which could make the individual studies prone to selection bias, as those who have more interest, awareness and are younger, tend to participate more compared to lower involvement of the elderly with minimal digital literacy and lower educational attainment, which may mask the true picture of vaccination status. Despite that, this method has the advantage of a higher reach of the intended sample, and it gained higher popularity during the COVID-19 pandemic as a result of emphasis on social distancing measures. Secondly, few studies specifically addressed vaccine uptake among older adults aged ≥ 65, or reported vaccine uptake estimates for that age group when the study included the whole adult population. In the current review, we included studies that report uptake for age groups starting at the age of 50 years old and above as a surrogate for ≥65 years old individuals in order to have better coverage for the elderly age group and overcome the scarcity of studies covering this important risk group. In line with that, most studies cover adults in general and provide estimates for the whole population without age-stratification for the elderly population within the study, which makes it difficult to extrapolate their results to different age strata of the intended population. Thirdly, the predictors presented in these studies usually emerge from a cross-sectional design, which lacks causal inference, and a few individual studies reported adjusted estimates for uptake predictors, raising the potential for confounding within these estimates; therefore, it is crucial for future studies to ensure that adjustments are applied for the included factors to account for potential confounders.

Further challenges encountered during this review include a lack of studies reporting on SIV specifically for certain chronic diseases in the post-COVID-19 vaccine era, such as cancers and liver diseases. These conditions may have been represented in studies that grouped chronic diseases; these diseases usually affect a smaller subset of the population, indicating the need to conduct disease-specific studies. Some studies reported estimates for chronic diseases in a broad context without matching uptake for each specific disease, which may reflect the fact that most individuals suffer from multiple rather than a single disease. Even for other high-risk groups, the available evidence remains limited, with few studies published in non-indexed journals that may have uncertain peer-review standards. These studies were retained to provide insight into SIV among target populations, given the limited number of available reports. This addresses the importance of performing robust studies among these high-risk groups.

Moreover, it is worth noting that SIV uptake in GCC countries may differ between early and late COVID-19 vaccine rollouts. We included all studies that collected data from December 2020, corresponding to the initial rollout of the COVID-19 vaccine worldwide and the associated influx of information about vaccine introduction. The large-scale rollout of COVID-19 varies between countries, even within the same countries, and across different risk groups. Therefore, we included studies from a common starting point; in addition to the already noted limited number of studies from some GCC countries, this may have resulted in the selection of an early phase point of the included studies post-COVID-19 vaccine introduction. Nevertheless, this temporal variation underscores the need for time-series studies. As observed in another review, we also noted that most research conducted in Saudi Arabia, compared to other GCC countries, some countries even lack recent prevalence of SIV acceptance for both general and high-risk groups [14]. This may be due to Saudi Arabia having a substantially higher population than other GCC countries, and to the increasing number of HCWs and active researchers in the country. This emphasises the need for robust studies covering these gaps. Finally, these factors, including heterogeneity in uptake outcomes and the limited number of studies among some high-risk groups and GCC countries, hindered the ability to conduct meta-analysis or systematic reviews across the included studies.

### 4.2. Future Research

Future research should match self-reported data with actual uptake from EHRs in each setting over the last 12 months or other standardised period to improve data homogeneity and enable comparability between countries or high-risk groups with different locations or periods. There is a need for prevalence studies among the elderly, which need more emphasis due to population ageing in GCC countries. In addition to that, other high-risk groups that are not covered, like cancer patients in all included countries or other groups that were not covered extensively after the COVID-19 pandemic era, particularly for countries other than Saudi Arabia. An additional need is for SIV uptake in GCC countries among non-local individuals, as they constitute a large share of the population and may face language or information-related barriers that hinder their acceptance during annual campaigns. Time-series studies may be needed to assess temporal changes in SIV coverage following the pandemic, as observed in other regions.

Strategies to increase uptake among high-risk groups during yearly vaccination campaigns are warranted. Efforts shall be made to explore the effectiveness of newer methods that may help improve vaccine acceptance, such as mobile-based reminders that include seasonal recommendations and vaccine-related information, targeting high-risk groups through commonly adopted governmental digital channels like the Tawakalna app or the Sehhaty app in Saudi Arabia, and their equivalents in GCC countries. These can be assessed through comparative, longitudinal, or trial studies to inform policy decision-making regarding SIV in GCC countries.

## 5. Conclusions

Suboptimal uptake is noted among most high-risk groups in GCC countries. Risk and severity perceptions, along with physicians’ involvement, significantly affected vaccine uptake among the intended population. More emphasis should be placed on effective risk communication and action cue methods to enhance uptake among high-risk groups. Future research is needed to cover understudied areas like the elderly aged ≥ 65 years, cancer and other high-risk groups, particularly in GCC countries other than Saudi Arabia, post-COVID-19 vaccine release.

## Figures and Tables

**Table 1:** Summary of studies of seasonal influenza vaccine (SIV) uptake, acceptance and willingness to vaccinate in the Gulf Cooperation Council countries post-COVID-19 vaccine introduction.

Author (Year)	Country	Population/Risk Group	Study Design	Risk group Sample Size (Total Sample Size, if Different)	Data Collection Period	SIV Outcome Definition	Main SIV Finding (%)	Notes
Hibshi et al. (2022) [19]	Saudi Arabia	Pregnant women	CS (online)	1790	July 2022	Ever vaccinated	53.7%	SIV timing not specified
Alqahtani et al. [20]	Saudi Arabia-Qassim	Pregnant women	CS (PHCs)	277	May–July 2023	Ever vaccinated	34.7%	SIV timing not specified
Altheyab et al. (2025) [21]	Saudi Arabia-Qassim	Pregnant women	CS (MCH)	501	March–November 2024	Current pregnancy uptake	1.8%	
Almutairi et al. (2023) [22]	Saudi Arabia-Taif	Dialysis	CS (Dialysis units)	463	2022	Current year uptake and annual adherence	64.1% current; 47.3% annual adherence	
Aljuman et al. (2025) [23]	Saudi Arabia-Jeddah	AIRDs	CS (clinic-based)	219	March–August 2024	Ever vaccinated	53.4%	-SIV timing not specified -Vaccination history verified with EHR -81.3% of sample were female
Saad et al. (2025) [24]	Saudi Arabia-Dammam	RA and SLE patients	CS (clinic-based)	261	November 2024–February 2025	Last two seasons’ uptake	27.59%	
Shehab et al. (2023) [25]	Kuwait	Inflammatory Bowel Disease	CS (Gastroenterology Centre)	394	January 2022–April 2023	Received the SIV	20%	Unclear/not specified SIV timing
Almishrafi et al. (2024) [26]	Saudi Arabia-Riyadh	Asthma	EHR Review(PHCs)	2689	October 2023–October 2024	2023–2024 season uptake	18.67%	Vaccination data from national registries
Alekri et al. (2022) [27]	Bahrain	DM (types1&2)	CS (PHCs)	393	February 2024	Ever vaccinated	50.6%	SIV timing not specified
Alqifary et al. [28]	Saudi Arabia	DM (types1&2)	EHR review (13 diabetic centres)	709	2023	Annual vaccine adherence	34.83%	Uptake data from multicenter EHRs
Aldhahir et al. [29]	Saudi Arabia-Jeddah and Asser	DM (types1&2)	CS (endocrinology and IM clinics)	1556	December 2023–March 2025	Ever vaccinated	60.8%	-SIV timing not specified -Multicenter
Gosadi et al. (2023) [30]	Saudi Arabia-Jazan	Chronic diseases	CS (online)	249 CD (out of 825)	October–November 2022	Annual uptake	17.3%	
Al nafea (2024) [31]	Saudi Arabia-Riyadh	Chronic disease	CS (online)	241	September–November 2023	Uptake/intention during 2023 season; uptake during 2020–2022 seasons	43.2% during 2023; 10.8% during 2020–2022	Assesses SIV uptake post-COVID-19
Hassan (2023) [32]	Oman	High-risk admitted adults	Record review (tertiary-care hospital)	158	January–February 2024	Current season uptake after previous admission for eligible adults	4.4%	Assesses compliance with the inpatients’ vaccination policy
Majrashi et al. (2021) [33]	Saudi Arabia	Older adults (≥60 years)	CS (online)	386	December 2020–January 2021	Current season uptake	66.6%	Pre–mass COVID-19 rollout
Al nafea (2024) [34]	Saudi Arabia-Riyadh	adults (≥51 years)	CS (online)	148 (out of 848)	September–November 2023	Uptake or intention during 2023 season	41.9%	Assesses SIV uptake post-COVID-19
Alshahrani et al. (2023) [35]	Saudi Arabia	adults (≥55 years)	CS (online)	33 (out of 758)	2022	Last 12 months uptake	18.2%	Assess the uptake post-COVID-19
Aziz et al. (2024) [36]	Qatar	PHCs physicians	CS (Online)	190	October 2021	Last season	73.7%	32.1% with chronic disease
Almusawi et al. (2025) [37]	Bahrain	HCWs	CS (Online and in-person)	552	July–September 2024	Annual uptake	64.5%	Majority were physicians and nurses
Alkathlan et al. (2021) [38]	Saudi Arabia-Qassim	Physicians and nurses	CS (online among two hospital staff)	424	January 2021	Intention during 2021 season	79%	
Almutairi et al. (2024) [39]	Saudi Arabia	HCWs at MOH facilities	CS (online)	1273	September–October 2021	Last 12 months uptake	50.8%	

SIV: seasonal influenza vaccine; CS: Cross-sectional study; MCH: Maternity and Children’s Hospital; PHCs: primary healthcare centres; AIRDs: autoimmune rheumatic diseases; EHR: electronic-health records; RA: rheumatoid arthritis; SLE: systemic lupus erythematosus; DM: diabetes mellitus; CD: chronic disease; HCWs: healthcare workers; MOH: Ministry of Health. Non-specified outcome duration, like “vaccination history”, “previously vaccinated”, was renamed into “ ever vaccinated”; SIV outcome included in the table represents relevant finding of SIV status post-COVID-19 vaccine introduction; if vaccination year was not specified, the ever vaccination percentage was presented.

**Table 2:** Predictors, facilitators, and barriers reported specifically to seasonal influenza vaccine uptake among high-risk groups, and acceptance or hesitancy among studies including only high-risk groups.

Study Author (Year)	Population	Outcome *	Key Predictors	OR (95% CI)/aOR (95% CI) (if Applicable)	*p*-Value	Main Facilitators	Main Barriers
Hibshi et al. (2022) [19]	Pregnant women	Acceptance	NR	-	-	Acceptance: 73.2% with physician advice and 49.2% with Family advice; SIV benefits women 58.4%	Not effective 32.4%; ↑ risk 26.1%; ↑ severity 21.4%
Alqahtani et al. [20]	Pregnant women	Ever vaccinated	NR	-	-	NR	Fear of injection; safety concerns; bad experience; not necessary
Altheyab et al. (2025) [21]	Pregnant women	Current pregnancy uptake	Younger women	-	0.047	NR	NR
			Being HCWs	-	0.025		
			First pregnancy	-	0.007		
Almutairi et al. (2023) [22]	Dialysis patients	Annual adherence	Good knowledge	2.1 (1.4–3.2)	-	NR	Non-vax (for current year): safety concerns 21.8%; effectiveness doubts 15.1%; may worsen kidney disease 14.5%
			Perceived risk of death	2.6 (1.6–4.3)	-		
			Proactive action: asking GP; asking nephrologists	For GP: 2.3 (1.2–4.3); For nephrologist: 2.0 (1.2–3.4)	-		
Saad et al. (2025) [24]	SLE and RA	Ever vaccinated	Older age	-	0.046	NR	NR
			Believe SIV is safe and effective	-	<0.001		
			SIV is the best way to prevent flu complications	-	0.001		
			Know SIV rec. for CDs	-	<0.001		
			Doctor advise	-	0.015		
Almishrafi et al. (2024) [26]	Asthma	2023–2024 season uptake	Aged 50–64 vs. younger or older	-	0.002	NR	NR
			Pneumococcal vaccine uptake	-	0.026		
			COVID-19 vaccine uptake	-	0.001		
			Prescribed a high dose of corticosteroid	-	0.001		
Alekri et al. (2022) [27]	Diabetes	Ever vaccinated	Female	1.8 (NR)	0.002	Vaccinated: 72.9% physician advice	Non-vax: 40.2% influenza is mild
			Intermediate education	3.3 (NR)	0.018		
			Longer time with DM	2 (NR)	0.018		
			Knowledge of flu and SIV	2 (NR)	<0.001		
Al nafea (2024) [31]	Chronic disease	No uptake during current season	Info from untrusted sources	1.49 (1.160–1.902)	-	Vaccinated: HCWs’ advice; personal health concerns; frequent RTIs; easy accessibility	Non-vax: uncomfortable without reason; effectiveness doubts; insufficient info on SIV need; no need for SIV; safety concerns
			Immunodeficiency patients	3.04 (1.05–8.81)	-		
			DM patients	0.64 (0.43–0.94)	-		
			Previous SIV during the pandemic	0.23 (0.095–0.55)	-		
Majrashi et al. (2021) [33]	Older adults (≥60 years)	Last 5 years regular uptake	Male	-	0.004	Vaccinated: 59.1% doctor advice; social media advice	Non-vax: flu is mild; effectiveness doubts
			Married	-	0.013		
			≥Secondary school education.	-	0.001		
			Living with a partner	-	0.016		
			High socioeconomic.	-	0.006		
			Residing in rural areas	-	0.001		
			History of flu-related hospital admission	-	0.049		
Aziz et al. (2024) [36]	PHCs physicians	Last season’s uptake	≥44 years	-	0.004	99% Free provision; >95% knowledge of SIV recommendation; >91% sufficient knowledge of flu	92.6% believe they can get the flu even with SIV; 29.5% prior side effects; 18.9% hard to take every year.
			Male	-	0.002		
			≥11 years of PHCs experience	-	0.005		
Alkathlan et al. (2021) [38]	HCWs (physicians and nurses)	Intention to vaccinate in the following (2021) season	>40 years	2.48 (1.03–5.96)	-	NR	NR
			Female	1.87 (1.06–3.29)	-		
			Non-Saudi	3.13 (1.85–5.29)	-		
			Being a nurse	3.12(1.69–5.56)	-		
Almutairi et al. (2024) [39]	HCWs	Past 12 months uptake	Vaccinated (last 5 years)	2.19 (1.49–3.22)	-	NR	NR
			Positive attitudes toward SIV	1.51 (1.09–2.09)	-		
			SIV workplace access	2.35 (1.56–3.52)	-		
			Know SIV rec. for HCWs.	1.68 (1.31–2.14)	-		
			Last year SIV activities	1.95 (1.47–2.58)	-		
			Low perceived flu risk	0.53 (0.32–0.86)	-		

Predictors, facilitators and barriers (explicitly mentioned as barriers in the study) reported specifically to seasonal influenza vaccine acceptance, uptake or intention or hesitancy among studies including only high-risk groups are reported in the table. OR (95% CI)/aOR (95% CI): Odd ratio/adjusted odd ratio (95% confidence interval); NR: not reported or specified for influenza vaccine; SIV: seasonal influenza vaccine; GP: general physician; Non-vax: not vaccinated; CDs: chronic diseases; DM: diabetes mellitus; HCWs: healthcare workers; RTIs: respiratory tract infections; PHCs: primary-healthcare centres; SLE: systemic lupus erythematosus; RA: rheumatoid arthritis. If OR (95%CI) was reported in the included studies, it was presented in the table; if not, the *p*-value was presented. * Outcomes presented here may vary from the outcomes presented in Table 1 for the same study.

## Data Availability

No new data were created or analyzed in this study. Data sharing is not applicable to this article..

## Abbreviations

The following abbreviations are used in this manuscript:

AIRDs	Autoimmune Rheumatic Diseases
CD	Crohn’s Disease
CDs	Chronic Diseases
CI	Confidence Interval
COVID-19	Coronavirus Disease 2019
COPD	Chronic Obstructive Pulmonary Disease
CS	Cross-Sectional study
DM	Diabetes Mellitus
DMARDs	Disease-Modifying Antirheumatic Drugs
GCC	Gulf Cooperation Council
GP	General physician
HBM	Health Belief Model
HCWs	Healthcare Workers
ICU	Intensive Care Unit
IM	Internal Medicine
MCH	Maternity and Children’s Hospital
MoH	Ministry of Health
NR	Not reported
OR	Odd Ratio
PHCs	Primary Healthcare Centres
RA	Rheumatoid Arthritis
RTIs	Respiratory Tract Infections
SI	Seasonal Influenza
SIV	Seasonal Influenza Vaccine
SLE	Systemic Lupus Erythematosus
UAE	United Arab Emirates
UC	Ulcerative Colitis
USD	United States Dollars
US	United States
WHO	World Health Organization
